# Early-onset thrombocytopenia, severe polyarthritis, and severe Β cell impairment in a Down syndrome patient

**DOI:** 10.1186/1939-4551-8-S1-A103

**Published:** 2015-04-08

**Authors:** Ana Paula Willy-Fabro, Ana Carolina Rozalem, Tatiane Pavan-Ramos, Cláudio Len, Juliana Themudo Lessa Mazzucchelli, Beatriz Costa Carvalho

**Affiliations:** 1 Federal University of Sao Paulo, Brazil

## Background

Down syndrome is the most common genetic disease and is associated with an increased frequency of congenital cardiac and gastrointestinal defects, hematological disorders, autoimmune diseases and immunodeficiency. Decreased numbers of T and B lymphocytes, particularly naive B cells, have been reported in these patients, although a significant association between lymphocyte subpopulation counts and the frequency of infections or the presence of autoimmunity was not found.

## Methods

Describe a case through retrospective review of clinical and laboratory data.

## Results

LAM, female, 5 years old, was born full term, adequate weight, and diagnosed with Down syndrome. She presented neonatal sepsis and thrombocytopenia that did not resolved after antibiotic use and required treatment with corticosteroids and immunoglobulin. At 20 months, she was admitted because of epistaxis and severe thrombocytopenia (1,000/mcl) which remitted only after three cycles of rituximab. At this point, she developed polyarticular arthritis that progressed to severe mobility restriction of hands, elbows and knees. From age 3 years onwards, she had recurrent episodes of sinusitis and was admitted four times due to pneumonia and wheezing. She has also been treated for hypothyroidism and gastroesophageal reflux disease. Cardiac and ophthalmological assessments were normal. Evaluation of the immune system when she was 4 years old showed very low immunoglobulin levels (IgA<7mg/dL, IgG=138mg/dL, IgM=12mg/dL, IgE=2kU/L) as in agammaglobulinemia and no specific antibody response to varicella zoster virus or measles vaccine. There were normal numbers of neutrophils. T lymphocytes and NK cells were CD3+:1,996/mm^3^, CD4+:1,181/mm^3^, CD8+:660/mm^3^, CD56+:594/mm^3^/ CD16+:555/mm^3^, respectively. B lymphocyte number was low (159/mm^3^ – 4,6%), with a quite low percentage of naïve (CD19+CD27-IgD+:0.13%) and absent memory B cells (CD19+CD27+IgD+:0%, CD19+CD27+IgD-:3.16%), with a high number of atypical memory B cells (CD19+CD27-IgD-:99.6%). She has been treated with methotrexate and leflunomide with articular improvement, besides immunoglobulin replacement therapy (0.5g/kg every 2 weeks).

## Conclusions

The early-onset hematological disorder, the severe autoimmune disease and the significant disturbance of B lymphocyte subpopulation, shown in this patient, accentuate how immunodysregulation significantly contributes to increase morbidity in Down syndrome.

## Consent

Written informed consent was obtained from the patient for publication of this abstract and any accompanying images. A copy of the written consent is available for review by the Editor of this journal.

